# Enhancing the Mechanical Properties and Water Permeability of Pervious Planting Concrete: A Study on Additives and Plant Growth

**DOI:** 10.3390/ma17102301

**Published:** 2024-05-13

**Authors:** Juan He, Shanhansu Xu, Guochen Sang, Yonghua Wu, Shuang Liu

**Affiliations:** 1 College of Materials Science and Engineering, Xi’an University of Architecture and Technology, Xi’an 710055, China; 13920966987@163.com (S.X.); wuyonghua@xauat.edu.cn (Y.W.); 17792799505@163.com (S.L.); 2 School of Civil Engineering and Architecture, Xi’an University of Technology, Xi’an 710048, China; sangguochen@xaut.edu.cn

**Keywords:** pervious concrete, water permeability, mechanical properties, sustainable building materials, plant growth, enhancement

## Abstract

Pervious planting concrete (PPC) is in line with the concept of ecological environmental protection. However, due to its own porous structure, it is difficult to obtain excellent mechanical properties and water permeability at the same time, which hinders its promotion and application. In this paper, natural gravel (NG), ordinary Portland cement (OPC), polyvinyl alcohol latex powder (PVAP) and polycarboxylate superplasticizer (PS) were used to prepare the PPC, and its mechanical properties and water permeability were studied. Three kinds of plants were planted in the PPC and their planting properties were studied. At the same time, the effect of *Bacillus* on the planting properties was studied. The results show that when the water–binder ratio (W/B) was 0.28 and the PVAP content was 0.8%, both the mechanical properties and water permeability of the PPC were optimal. The compressive strength and permeability coefficient were 14.2 MPa and 14.48 mm/s, respectively. The mechanical properties and water permeability of PPC prepared with 10~20 mm NG were better than those prepared with 5~10 mm NG. Among the three plants, the germination rate and growth of Elymus dahuricus Turcz (EDT) were the best. The incorporation of *Bacillus* can optimize its planting properties and promote the effective combination between plants and the PPC substrate.

## 1. Introduction

The growth of urbanization brings global challenges such as stormwater management, urban heat islands, and the loss of green space. Sustainable building materials, such as pervious planting concrete, which supports water permeability and urban vegetation, can help alleviate these issues. Moreover, sustainable building materials can be used in different types of concrete to reduce environmental impact, improve the relationship between humans and ecology, and promote the health and well-being of building occupants [1,2].

Pervious planting concrete (PPC) was first proposed in Japan in the 1990s. It is a concrete structure prepared with a coarse aggregate and cementitious binder [3,4]. Its structure is similar to that of “Sachima”, which can make plant seeds take root and germinate in the pores or on the surface. The roots grow in the pores of the concrete and merge with the concrete structure to form a whole. PPC plays an effective role in protection and landscapes, and has been applied well in slope protection engineering [5,6,7]. PPC shows good water permeability due to its large pore structure. It can make rainwater penetrate into the ground quickly, clean up the accumulation of rainwater on the surface in time, and replenish groundwater resources. PPC plays an important role in improving the exchange properties of water and soil on the surface, conserving water, and improving water quality [8,9,10].

Scholars have studied some properties of PPC. Chen Wei et al. studied the effect of silica fume and diatomite on the mechanical properties of PPC prepared with high-content red mud [11]. It was found that an appropriate content of silica fume and diatomite could make its structure denser and provide excellent pore characteristics for PPC. The optimal mechanical properties reached about 10 MPa. Gong Chenchen et al. studied the effect of carbamide content on the water permeability of PPC [12]. The results showed that the reaction between CO_3_^2−^ introduced by carbamide hydrolysis and Ca^2+^ introduced by cement hydration improved the fluidity of the cement slurry. The cement slurry can be more evenly wrapped on the surface of the aggregate, forming well-connected pores and improving the water permeability. However, while the water permeability was increased, the mechanical properties would be decreased. The results of Ariffin N. F. et al. showed that with an increase in cementitious materials, the porosity and water permeability of PPC decreased, and the compressive strength increased [13].

Aggregate size is a factor affecting the properties of PPC. Chen Jingguo et al. used 10~30 mm and 20~40 mm aggregate and different water–binder ratios (W/B) to prepare PPC. The results showed that the compressive strength of the PPC prepared with 10~30 mm aggregate was slightly lower than that prepared with 20~40 mm aggregate. There was no obvious linear relationship between mechanical properties and W/B, and there was an optimal W/B in different mix ratios [14]. Huang Yamei et al. compared the compressive strength of PPC prepared by aggregate with three different sizes of 5~20 mm, 16~31.5 mm and 20~40 mm. It was found that the larger the aggregate size, the larger the internal pores formed in the concrete, and the lower the compressive strength [15]. Liu Ying et al. conducted a comparative study on W/B, target porosity, and aggregate size. It was found that the influence of aggregate size on mechanical properties was slightly lower than the target porosity, but higher than the W/B [16].

Omka et al. studied the compatibility of plants with PPC, and found that the substrate could provide a good environment for the plants growing on it, realizing the ecological concept of coexistence [17]. Due to its good planting properties, it was well applied in ecological slope protection. Wu Yanyou et al. studied the compatibility between *Orychophragmus violaceus* and PPC, and found that its roots could be integrated with PPC. Compared with the control group growing in soil, there were more lateral roots and fibrous roots around the taproot of *Orychophragmus violaceus* growing in PPC [18]. Di Li et al. studied and compared *Cynodon dactylon* (L.) Pers (CDP), *Agrostis stolonifera*, and *Sasa argenteostriatus* [19]. The growth of the three plants under natural conditions was better than that in concrete. The plants on concrete prepared with large-size aggregates showed better adaptability than plants on concrete prepared with small-size aggregates. Therefore, the selection of plants is also an important factor in the preparation of PPC, and the size of the aggregate will also affect the development of the plant.

The planting properties of PPC can be improved by other means [20]. *Bacillus* is a kind of bacteria that can produce spores. It has the functions of water purification, soil improvement and plant growth promotion. The spores produced by *Bacillus* are alkali- and drought-tolerant. This is very consistent with the concept of PPC [21,22,23]. Poly-γ-glutamic acid, the metabolite of *Bacillus licheniformis* and *Bacillus subtilis*, can be used to prepare environmentally friendly degradable polymer materials [24,25]. *Brevibacillus laterosporus* can convert propyl 4-hydroxybenzoate into gentisic acid, which is used to treat industrial wastewater and degrade toxic substances in the environment [26]. Extracellular polysaccharide and extracellular protein produced by *Paenibacillus polymyxa* can be used as flocculants to treat wastewater [27].

Although some scholars have carried out research on the mechanical properties and water permeability of PPC, it is difficult for them to obtain good compressive strength and excellent permeability coefficient at the same time. In order to improve these properties, natural gravel (NG) and ordinary Portland cement (OPC) were used to prepare PPC, and polyvinyl alcohol latex powder (PVAP) and water reducer were added to optimize its properties. The mechanical properties and water permeability of PPC were studied and discussed. Subsequently, three different plants were selected for planting in the PPC. *Bacillus* was added to study and improve the planting properties. It is expected to provide a theoretical basis for the application and promotion of PPC.

## 2. Materials and Methods

### 2.1. Materials

The cementitious binder was OPC with a strength grade of 42.5. The initial and final setting times were 170 and 275 min. The 3-day and 28-day compressive strengths were 38.4 MPa and 58.4 MPa. The aggregate was NG with sizes of 5~10 mm and 10~20 mm, respectively. The main physical properties of NG are shown in Table 1. The cold-water instant PVAP was the 2488 type produced by Jinzhou Baoyi Building Materials Technology Co., Ltd. (Jinzhou, China) and its mesh number was 160 mesh. The water reducer was a polycarboxylate superplasticizer (PS) with high performance. Its recommended content was 0.8~1%, and the water reduction ratio was approximately between 25 and 30%. The water was tap water, which is in line with Chinese national building materials industry standard “Water standard for concrete” (JGJ63-2006) [28]. The plants were CDP, Elymus dahuricus Turcz (EDT), and Festuca elata Keng ex E. Alexeev (FKEA). The *Bacillus* was compound *Bacillus*. Its effective viable bacteria were higher than 20 billion/g, and the enzyme activity was higher than 2000 U/g.

### 2.2. Mixing Proportion Design

On the basis of preliminary exploratory tests, it was found that when the binder–aggregate ratio (B/A) of PPC was 0.2, the amount of cement slurry exactly wrapped around the surface of the NG and formed well-connected pores. When the B/A was larger, the cement slurry would block the pores and sink to the bottom. When the B/A was smaller, the amount of cement slurry could not completely wrap around the surface of the aggregate. Therefore, the B/A was fixed at 0.2. Two kinds of NGs with different sizes, six kinds of W/B and six PVAP contents were used to prepare PPC and the content of PS was fixed at 1.0%. The effects of aggregate size, W/B, and PVAP content on the mechanical properties and water permeability of PPC were studied. The specific mixing proportion is shown in Table 2. Subsequently, the three selected plants were planted on the prepared PPC substrate. 

### 2.3. Experimental Methods

#### 2.3.1. Mechanical Properties

The mechanical properties were studied according to Chinese national building materials industry standard “Standard for test methods of physical and mechanical properties of concrete” (GB/T 50081-2019) [29]. OPC, PS, PVAP, and water were poured into the concrete mixer and stirred for 30 s. Then, natural aggregates were added and stirring was continued for 120 s. The mixed PPC concrete was cast into 150 mm cubic molds two times, and the loading thickness was roughly the same each time. The specimens were tamped with a tamping rod. Tamping was carried out evenly from the edge to the center along the spiral direction, and each layer was rammed 29 times. After 7 days and 28 days of standard curing, the mechanical properties were measured on three specimens for each studied composition. The requirements for mechanical properties in the national standard are shown in Table 3 [29].

#### 2.3.2. Water Permeability

**Figure 1:**
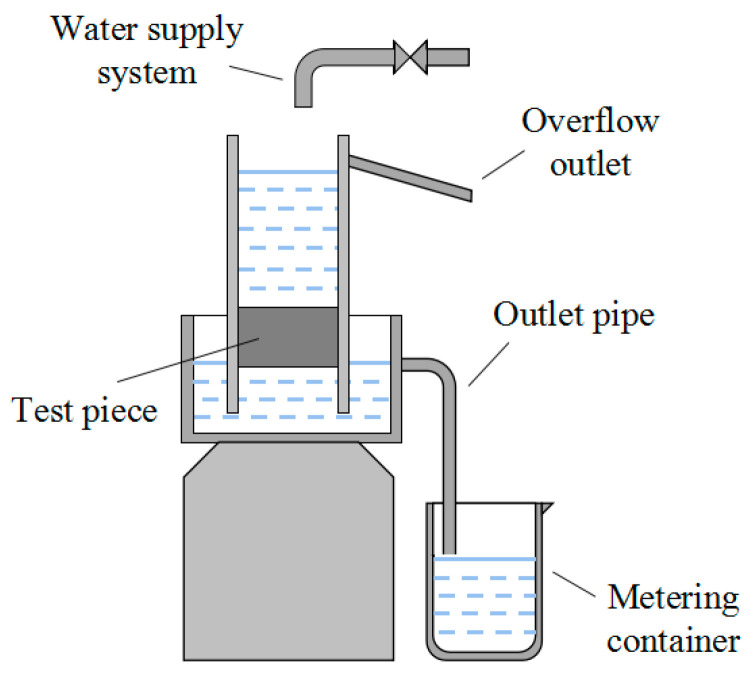
The test device for water permeability.

The water permeability was carried out according to Chinese national building materials industry standard “Pervious concrete” (JC/T 2558-2020) [30]. The mixing and forming process was the same as that of the mechanical properties. Three cylindrical specimens with a size of φ100 mm × 50 mm were cast. After 27 days of standard curing, the specimens were soaked in water for 24 h at about 15 °C, and then the water permeability was tested. The test device for water permeability is shown in Figure 1.

The water permeability is characterized by the permeability coefficient, and the calculation formula is shown in Formula (1):

(1) $$K_{15}=\frac{QL}{AHt}$$

In the formula, *K*_15_ is the permeability coefficient of the specimen when the temperature is 15 °C (mm/s); *Q* is the amount of water flowing out in time t seconds (mm^3^); *L* is the thickness of the specimen (mm); *A* is the upper surface area of the specimen (mm^2^); *H* is the water level difference (mm); and *t* is the time (s).

In the national standard, the permeability coefficient is divided into different grades, and the specific requirements are shown in Table 4 [30].

#### 2.3.3. Planting Properties

**Figure 2:**
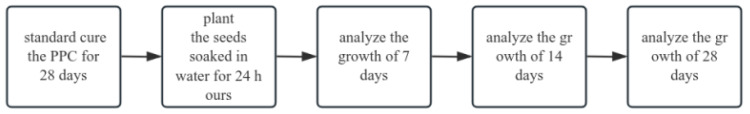
Flow chart for planting properties of PPC.

The mixing proportions with the best mechanical properties and water permeability were selected to prepare the PPC cubic substrates with 100 mm sides. The PPC substrate was standard-cured for 28 days and then covered with 2 cm thick soil. The seeds of CDP, EDT, and FKEA were soaked in water for 24 h, and then were spread in the soil on the surface of the PPC substrate. Next, growth analysis was performed at 7 days, 14 days, and 28 days. The adaptability of PPC with three plants was studied to determine whether it was suitable for growing on PPC. Planting properties are characterized by germination rate, coverage, and growth height. The specific test process is shown in Figure 2. The one with the best growth conditions was selected and mixed with the compound *Bacillus* to study its effect on planting properties.

## 3. Results and Discussion

### 3.1. Mechanical Properties of PPC

#### 3.1.1. Effect of W/B on Mechanical Properties 

**Figure 3:**
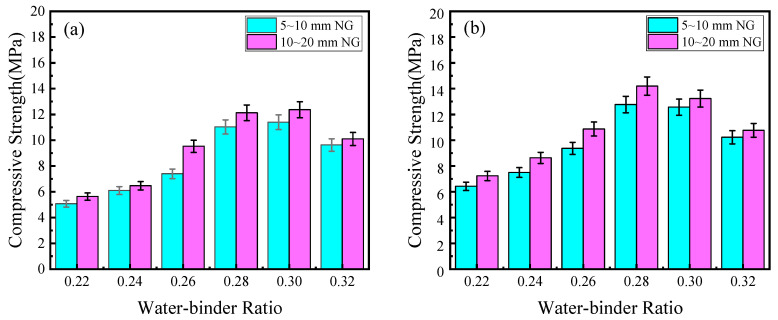
The effect of W/B on the compressive strength of PPC: (**a**) 7 days, (**b**) 28 days (the PVAP content is 0.8%).

The effect of W/B on the mechanical properties of PPC is shown in Figure 3. It can be seen that with the increase in W/B, the 7-day and 28-day compressive strengths increased first and then decreased. The variation in PPC prepared with 5–10 mm NG was similar to that of PPC prepared with 10–20 mm NG. It can be seen from Figure 3a that all compressive strengths exceeded the national standard requirement for 7-day compressive strength. When the W/B changed from 0.22 to 0.28, the 7-day compressive strength of the PPC prepared with small-size NG and large-size NG increased by 125% and 119%, respectively. It can be seen from Figure 3b that when the W/B changed from 0.22 to 0.28, the 28-day compressive strength gradually increased, and the PPC prepared with small-size NG and large-size NG increased by 99% and 96%, respectively. When the W/B was 0.28, the 28-day compressive strength of the PPC prepared with small-size NG and large-size NG was the highest, with 12.8 MPa and 14.2 MPa, respectively. The 28-day compressive strength required by the national standard is 10 MPa, and the compressive strength values of the PPC prepared with two types of NG were 28% and 42% higher than the national standard requirements, respectively.

**Figure 4:**
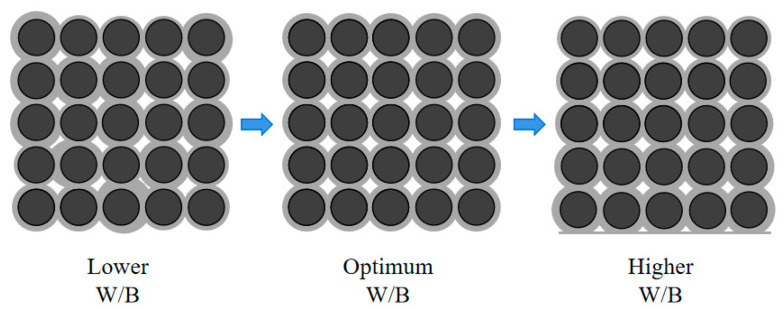
The effect of W/B on the internal structure and the pores of PPC.

The effect of W/B on the internal pore structure of PPC and the coating of the aggregate with cement slurry are shown in Figure 4 (See Figure 9). As can be seen, when the W/B was low, the amount of water was low and the mixture was too dry, so it could not be evenly coated on the surface of the gravel particles. The pores formed in PPC were of different sizes and unevenly distributed. Furthermore, cement particles were not sufficiently hydrated, and the bonding properties between aggregates were reduced. Therefore, when the concrete was compressed, the interface between the aggregate and the cement paste was easy to crack, resulting in damage to the overall structure. With the increase in W/B, the amount of cement paste that underwent a hydration reaction increased, which could improve the coating of the cement slurry on the aggregate and optimize the pore structure. When the W/B was optimal, PPC showed good workability and a uniform coating layer, which improved the bonding and mechanical properties. However, as shown on the right side of Figure 4 (See the last two figures of Figure 9), when the W/B was too high, in addition to the amount of water required to meet the normal hydration of cement, excess water formed blisters and pores in the PPC. In addition, slurry would sink to the bottom of the PPC, and the cement slurry of the upper part would be less than the lower part, which was not conducive to its mechanical properties. As a result, the actual effective bearing section of PPC was reduced, and the compressive strength was reduced [10,31].

#### 3.1.2. Effect of PVAP on Mechanical Properties

**Figure 5:**
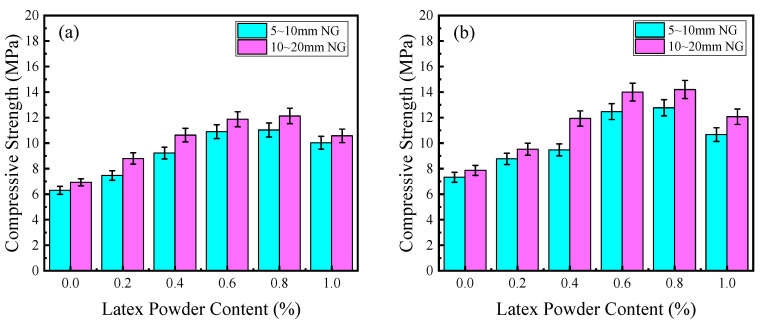
The effect of PVAP on the compressive strength: (**a**) 7 days, (**b**) 28 days (the W/B is 0.28).

The effect of PVAP on the mechanical properties of PPC is shown in Figure 5. It can be seen that with the increase in PVAP, the 7-day and 28-day compressive strengths first increased and then decreased. The variation of PPC prepared with 5~10 mm NG was similar to that of PPC prepared with 10~20 mm NG. When PVAP content varied from 0 to 0.8%, the 7-day compressive strength of PPC prepared with small-size NG and large-size NG increased by 75% and 76%, and the 28-day compressive strength increased by 63% and 81%, respectively. When PVAP was 0.8%, the 28-day compressive strength of the PPC prepared with small-size NG and large-size NG reached the maximum, which was 12.8 MPa and 14.2 MPa, respectively. When PVAP was 0.6%, compressive strength was slightly lower than that of 0.8%. In addition, the mechanical properties were all higher than the previous scholars’ research results or national standards [11,29].

**Figure 6:**
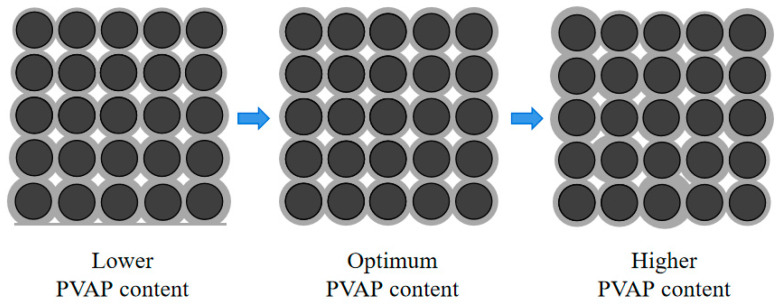
The effect of PVAP content on the internal structure and pores of PPC.

The effect of PVAP on the internal pore structure of PPC and the coating of the aggregate by cement slurry is shown in Figure 6 (See Figure 11). The incorporation of PVAP mainly affected the viscosity of the cement slurry and the workability of the PPC mixture. In the case of no PVAP or a low content of PVAP (less than 0.4%), the cement slurry presented low viscosity and was easy to deposit at the bottom. With the increase in PVAP (from 0.6% to 0.8%), the viscosity of the cement slurry increased, and the aggregate particles could be better wrapped. Also, the pore size and pore distribution were more uniform. The overall stress of PPC was uniform and would not cause stress concentration and damage [32,33]. In addition, the dehydration and film formation of PVAP in the concrete could improve the adhesive property between the cement slurry and aggregate and make the microstructure of the PPC more dense. So, the compressive strength of the PPC was improved. However, when the content of PVAP was too high, the cement slurry was too viscous and could not coat the aggregate particles well to form a uniform “Sachima” structure. Also, excessive PVAP would wrap some cement particles, affecting their hydration, which was not conducive to the development of adhesive properties. The overall stress was uneven and more prone to damage, resulting in a decrease in compressive strength.

**Figure 7:**
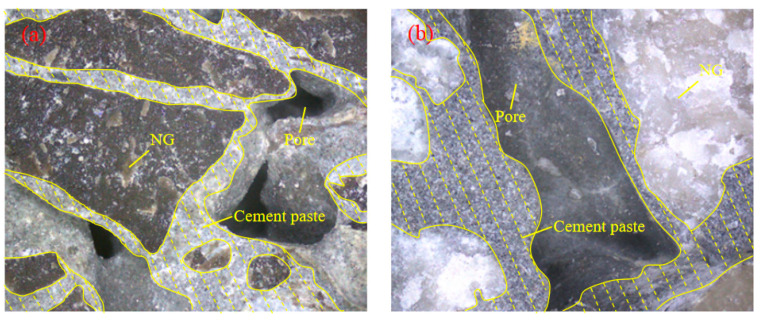
The cement paste contact layer of PPC: (**a**) 5~10 mm NG and (**b**) 10~20 mm NG (the enclosed dotted line is the cement paste; the remainder is NG and pores).

Furthermore, according to Figure 3 and Figure 5, it can be seen that under the same W/B, PVAP content, and age, the compressive strength of PPC prepared with 10~20 mm NG was higher than that prepared with 5~10 mm NG, and the difference was between 0.1 MPa and 2.5 MPa. The encapsulation of aggregate by cement slurry is shown in Figure 7. It can be found that the contact layer of the cement slurry between the gravel particles of PPC prepared with 10~20 mm NG was thicker than that of PPC prepared with 5~10 mm NG, and a more effective bond could be formed. Compared with 5~10 mm NG, the surface area of 10~20 mm NG was relatively small, and the amount of cement slurry wrapped in NG was less, so the cement slurry of the contact layer would be thicker. This improved the mechanical properties of PPC with 10~20 mm NG.

### 3.2. Water Permeability of PPC

#### 3.2.1. Effect of W/B on Water Permeability

**Figure 8:**
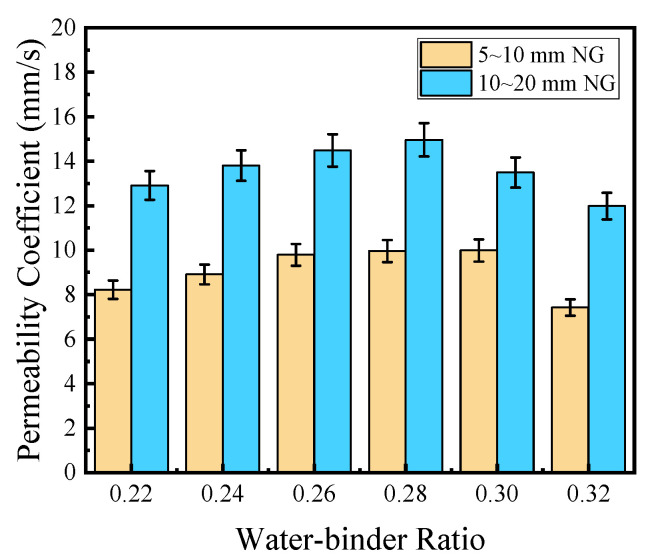
The effect of W/B on the water permeability of PPC (The PVAP content is 0.8%).

The effect of W/B on the water permeability of PPC is shown in Figure 8. It can be seen that with the increase in W/B, the permeability coefficient first increased and then decreased. The change in PPC prepared with 5~10 mm NG was similar to that of PPC prepared with 10~20 mm NG. The permeability coefficient of the latter was higher than the former. When the W/B was from 0.22 to 0.30, the permeability coefficient of PPC prepared with 5~10 mm NG increased by 22%, and it reached the maximum value of 9.99 mm/s when the W/B was 0.30. The permeability coefficient varied little when W/B was 0.26–0.30. When the W/B was from 0.22 to 0.28, the permeability coefficient of PPC prepared with natural 10~20 mm NG increased by 16%, and it reached the maximum value of 14.96 mm/s when the W/B was 0.28. It far exceeded the requirements of the national standard for water permeability coefficient grade K8.

**Figure 9:**
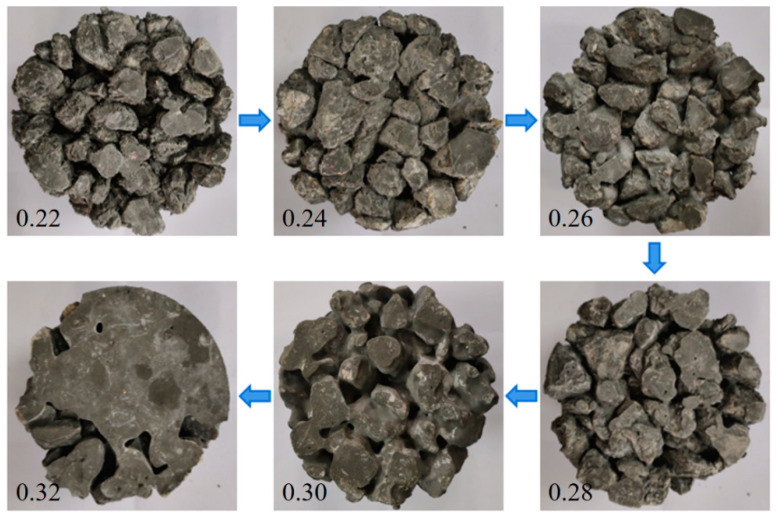
Effect of W/B on cement-slurry-coating aggregate (upside-down image of PPC; 10–20 mm NG).

The effect of W/B on the encapsulation of aggregate by cement slurry is shown in Figure 9. When the W/B was low, the cement slurry was dry and thick, and the cement slurry could not coat or bond with the aggregate well. The pores formed were unevenly distributed and the connectivity was low. So, the water permeability was poor. With the increase in W/B, the amount of cement paste increased, which could improve the coating of the cement slurry on aggregate and optimize the pore structure. The pores formed were interconnected, and could effectively and quickly discharge water [34]. So, the water permeability of PPC was improved. When W/B was too high, the volume of cement pastes increased and the viscosity decreased. Although it was conducive to the uniform mixing of PPC, the slurry was easy to sink, and the slurry layer that formed at the bottom blocked the pores and was not conducive to water permeability [35]. The law of cement-wrapped aggregates caused by changes in the water–cement ratio of PPC prepared with 5–10 mm NG was similar to that of PPC prepared with 10–20 mm NG. 

#### 3.2.2. Effect of PVAP on Water Permeability

**Figure 10:**
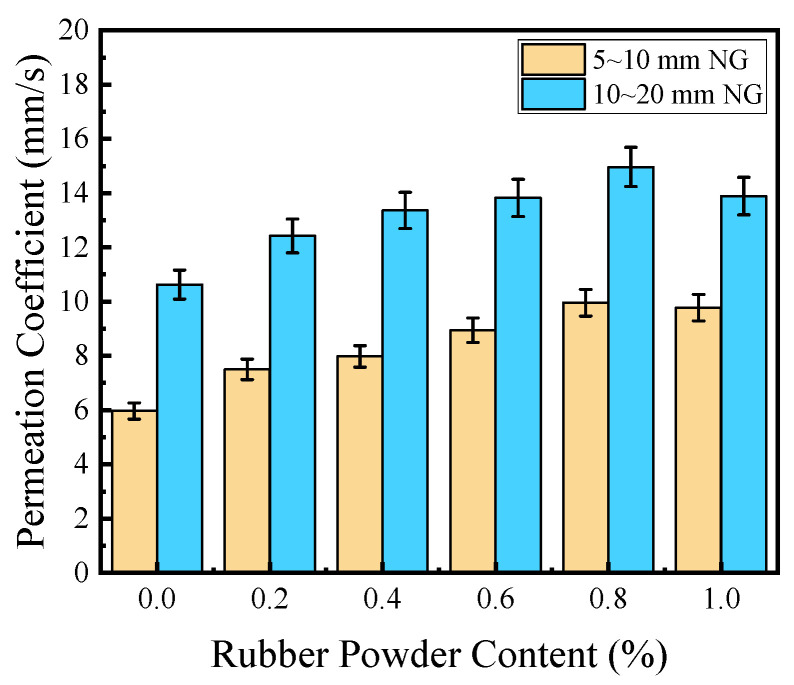
The effect of PVAP on the water permeability of PPC (the W/B is 0.28).

The effect of PVAP on the water permeability of PPC is shown in Figure 10. It can be seen that with the increase in PVAP content, the permeability coefficient first increased and then decreased. The variation in PPC prepared with 5~10 mm NG was similar to that of PPC prepared with 10~20 mm NG. For the former, when PVAP increased from 0 to 0.8%, and the permeability coefficient increased by 67%. When the PVAP was 0.8%, the permeability coefficient was the highest and it was 9.96 mm/s. For the latter, when PVAP increased from 0 to 0.8%, the permeability coefficient increased by 41%. When the PVAP content was 0.8%, the permeability coefficient reached the highest of 14.96 mm/s. In summary, the optimal value of PVAP content was 0.8%.

**Figure 11:**
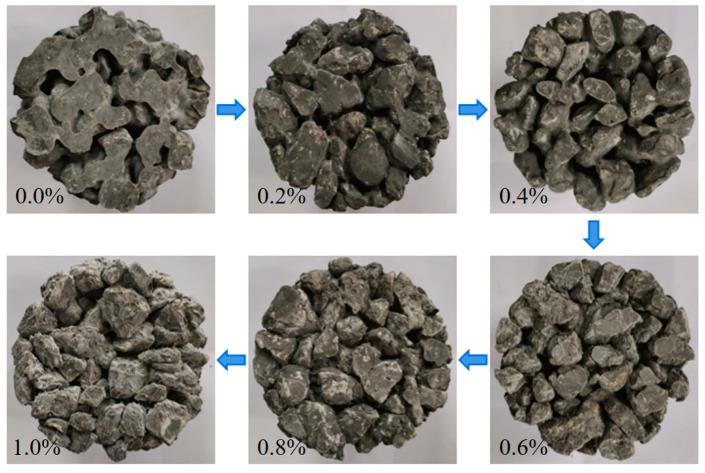
Effect of PVAP on cement slurry coating aggregate (upside-down image of PPC; 10–20 mm NG).

The effect of PVAP on the encapsulation of aggregate by cement slurry is shown in Figure 11. The incorporation of PVAP can increase the viscosity of the cement slurry and affect the workability of the PPC mixture. When no PVAP was added or the PVAP content was too low (less than 0.4%), the viscosity of the cement slurry was low and it was deposited easily at the bottom. With the increase in PVAP, the cement slurry viscosity increased, the slurry sinking phenomenon decreased, and the water permeability increased. As shown in Figure 11, when the PVAP was 0.6% and 0.8%, the aggregate was coated well by the cement slurry and the distribution of internal pores in PPC was uniform. The interconnection of the pores was good in the PPC and improved the water permeability [36]. When the content of PVAP was too high, as described earlier, the cement slurry was too viscous and could not coat the aggregate particles well. The pore distribution was uneven, and the connected pores were fewer. So, water permeability was reduced. The water permeability variation of 5–10 mm NG with the increase in PVAP content was similar to that of 10–20 mm NG.

### 3.3. Planting Properties of PPC 

#### 3.3.1. Effect of Plants on Planting Properties

**Figure 12:**
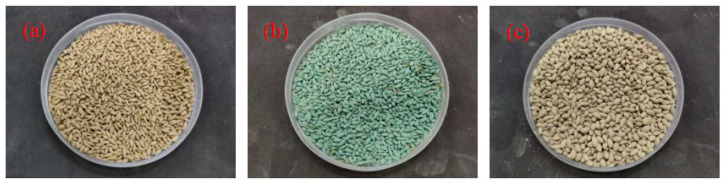
Three seeds (**a**) CDP; (**b**) EDT; (**c**) FKEA.

According to the above test results, the mechanical properties and water permeability of PPC prepared with 10~20 mm NG were better than those prepared with 5~10 mm NG. Therefore, the PPC prepared with 10~20 mm NG was selected as the substrate for the study of plant growth. The W/B was 0.28, and the PVAP was 0.8%. Figure 12 shows three seeds of CDP, EDT, and FKEA, which are suitable for growth under alkaline conditions. Here, 100 seeds of each plant were spread on the surface of the PPC substrate, and then covered with soil. They were watered regularly and the growth was observed.

**Figure 13:**
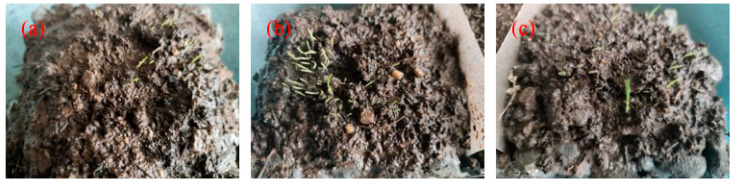
The growth of plants at 7 days: (**a**) CDP; (**b**) EDT; (**c**) FKEA.

The germination rate and growth height of three plants at 7 days under the same culture conditions are shown in Figure 13. It can be clearly observed that the germination rate of EDT was the highest. The new buds were dense, the growth height was up to 1 cm, and the growth conditions were good. The germination amount of FKEA was slightly less than that of EDT. Although some seeds grew no buds, the growth height was the same as that of EDT. The lowest germination rate was CDP, where only a few new buds germinated and the growth height was less than 1 cm.

**Figure 14:**
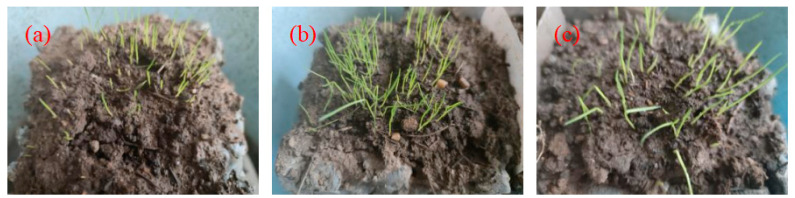
The growth of plants at 14 days: (**a**) CDP; (**b**) EDT; (**c**) FKEA.

**Figure 15:**
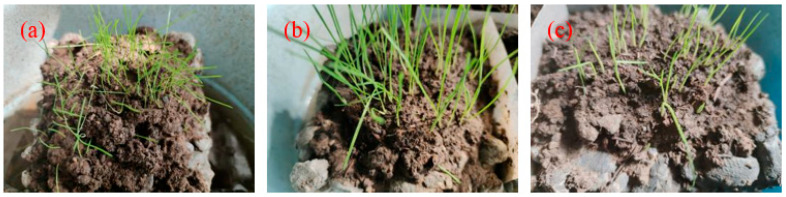
The growth of plants at 28 days: (**a**) CDP; (**b**) EDT; (**c**) FKEA.

The germination rate and growth height of three plants at 14 days under the same culture conditions are shown in Figure 14. Similar to 7 days, the growth of EDT was the best, followed by FKEA and CDP. The height of EDT was more than 3 cm, and some reached 5 cm. Although all three plants continued to produce new buds, plant coverage of FKEA and CDP was less than that of EDT. The growth of three different plants at 28 days are shown in Figure 15. The growth pattern was similar to that at 7 days and 14 days. The growth patterns of CDP and FKEA gradually became similar, and both were weaker than that of EDT. The plants were scattered, and the growth height was low, and even collapsed.

It can be concluded that the growth of EDT in the PPC substrate is better than that of CDP and FKEA, and the growth conditions are more suitable for this alkaline porous concrete environment. In the selection of plants, EDT should be the first choice for planting. It is also proved that the PPC substrate can present excellent planting performance, which can provide a good growth environment for plants.

#### 3.3.2. Effect of *Bacillus* on Planting Properties

**Figure 16:**
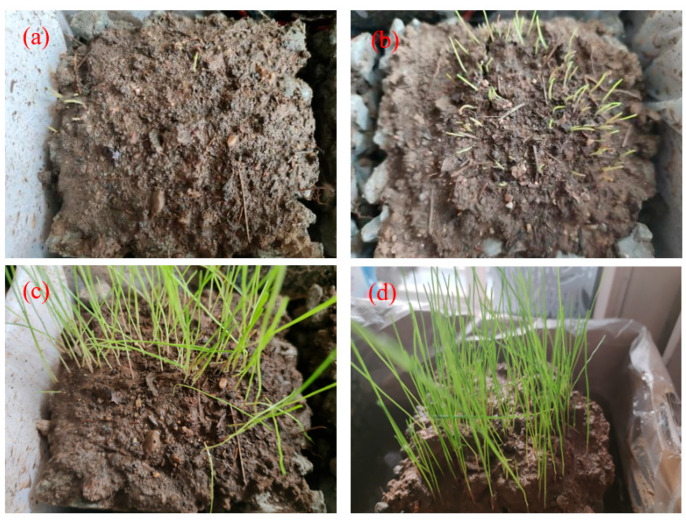
The growth of EDT and the growth of EDT with *Bacillus*: (**a**) EDT at 7 days; (**b**) EDT at 28 days (**c**) EDT with *Bacillus* at 7 days; (**d**) EDT with *Bacillus* at 28 days.

The effect of *Bacillus* on planting properties is shown in Figure 16. 

It can be seen clearly that under the same culture conditions, *Bacillus* greatly increased the germination ratio of EDT and promoted the growth rate of EDT. At 7 days, the growth height of EDT was about 1 cm, and that of EDT with *Bacillus* exceeded 1 cm, and with some reaching 2 cm. At 28 days, the growth height of EDT was about 9 cm, and that of EDT with *Bacillus* was slightly higher than 9 cm. The plant coverage area of EDT was about 50%, and that of EDT with *Bacillus* was more than 80%. The plants of EDT were evenly distributed on the substrate of PPC when *Bacillus* was added. *Bacillus* played a role in promoting the germination and growth of plants, which was conducive to improving the planting properties of PPC and promoting the organic integration between plants and PPC.

## 4. Conclusions

In the paper, the PPC was prepared with NG, OPC, PVAP, and PS, and its mechanical properties and water permeability were studied. CDP, EDT, and FKEA were planted in the PPC and their planting properties were studied. At the same time, the effect of *Bacillus* on the planting properties was studied. The following conclusions were drawn.

(1) With the increase in W/B or PVAP content, the mechanical properties of PPC increased first and then decreased. When the W/B was 0.28 and the PVAP content was 0.8%, the mechanical properties of the PPC prepared with 5~10 mm NG and 10~20 mm NG were optimal; 12.8 MPa and 14.2 MPa.
(2) With the increase in W/B or PVAP content, the water permeability of PPC increased first and then decreased. When the W/B was 0.28 and the PVAP content was 0.8%, the water permeability of the PPC prepared with 5~10 mm NG and 10~20 mm NG was optimal; 9.96 mm/s and 14.96 mm/s.
(3) The mechanical properties and water permeability of PPC prepared with 10~20 mm NG were better than those of PPC prepared with 5~10 mm NG. The effect of W/B or PVAP on the two was similar. The 10~20 mm NG was suitable for the preparation of PPC.
(4) Compared with CDP and FKEA, the germination rate and growth height of EDT were better under the same culture conditions, and it was more suitable for application on the PPC substrate. The addition of *Bacillus* was beneficial to the germination and growth of plants, which could improve the planting properties of PPC and promote the organic integration between plants and the PPC substrate.

Next, the following need to be studied further. A large-scale outdoor test of PPC will be carried out to study its performance development in different seasons and climatic conditions, laying the foundation for engineering applications. The water purification performance of PPC will be studied. PPC shows good characteristics for filtering and decomposing pollutants, which is of great significance for urban water cycle and environmental protection.

## Figures and Tables

**Table 1:** Main physical properties of NG.

Size (mm)	Apparent Density (kg/m^3^)	24 h Water Absorption Rate (%)	Bulk Density (kg/m^3^)	Water Content (%)	Mud Content (%)	Crush Index (%)
5~10	2761	0.69	1597	0.27	0.55	12.81
10~20	2743	0.81	1574	0.22	0.32	9.96

**Table 2:** Mixing proportion design of PPC.

Sample	Aggregate Size (mm)	NG (kg/m^3^)	Cement (kg/m^3^)	Water (kg/m^3^)	W/B	PVAP (%)	PS (%)
BS-1	5~10	1500	300	66	0.22	0.8	1.0
BS-2	5~10	1500	300	72	0.24	0.8	1.0
BS-3	5~10	1500	300	78	0.26	0.8	1.0
BS-4	5~10	1500	300	84	0.28	0.8	1.0
BS-5	5~10	1500	300	90	0.30	0.8	1.0
BS-6	5~10	1500	300	96	0.32	0.8	1.0
BL-1	10~20	1500	300	66	0.22	0.8	1.0
BL-2	10~20	1500	300	72	0.24	0.8	1.0
BL-3	10~20	1500	300	78	0.26	0.8	1.0
BL-4	10~20	1500	300	84	0.28	0.8	1.0
BL-5	10~20	1500	300	90	0.30	0.8	1.0
BL-6	10~20	1500	300	96	0.32	0.8	1.0
CS-1	5~10	1500	300	84	0.28	-	1.0
CS-2	5~10	1500	300	84	0.28	0.2	1.0
CS-3	5~10	1500	300	84	0.28	0.4	1.0
CS-4	5~10	1500	300	84	0.28	0.6	1.0
CS-5	5~10	1500	300	84	0.28	0.8	1.0
CS-6	5~10	1500	300	84	0.28	1.0	1.0
CL-1	10~20	1500	300	84	0.28	-	1.0
CL-2	10~20	1500	300	84	0.28	0.2	1.0
CL-3	10~20	1500	300	84	0.28	0.4	1.0
CL-4	10~20	1500	300	84	0.28	0.6	1.0
CL-5	10~20	1500	300	84	0.28	0.8	1.0
CL-6	10~20	1500	300	84	0.28	1.0	1.0

**Table 3:** Requirements for mechanical properties in the national standard.

Age (days)	Compressive Strength (MPa)
7	≥3
28	≥10

**Table 4:** The grade of permeability coefficient.

Grade of Permeability Coefficient	Permeability Coefficient (mm/s)
K0.5	≥0.5
K1	≥1
K2	≥2
K4	≥4
K6	≥6
K8	≥8

## Data Availability

Data are contained within the article.
